# The clinical utility window for acute kidney injury biomarkers in the critically ill

**DOI:** 10.1186/s13054-014-0601-2

**Published:** 2014-11-04

**Authors:** Azrina Md Ralib, John W Pickering, Geoffrey M Shaw, Martin P Than, Peter M George, Zoltán H Endre

**Affiliations:** Department of Anaesthesiology and Intensive Care, International Islamic University Malaysia, Kuantan, Pahang Malaysia; Department of Medicine, University of Otago, Christchurch, New Zealand; Intensive Care Unit, Christchurch Hospital, Christchurch, New Zealand; Emergency Department, Christchurch Hospital, Christchurch, New Zealand; Canterbury Health Laboratories, Christchurch, New Zealand; Department of Nephrology, Prince of Wales and Clinical School, University of New South Wales, High Street, Randwick, Sydney, NSW 2031 Australia

## Abstract

**Introduction:**

Acute Kidney Injury (AKI) biomarker utility depends on sample timing after the onset of renal injury. We compared biomarker performance on arrival in the emergency department (ED) with subsequent performance in the intensive care unit (ICU).

**Methods:**

Urinary and plasma Neutrophil Gelatinase-Associated Lipocalin (NGAL), and urinary Cystatin C (CysC), alkaline phosphatase, γ-Glutamyl Transpeptidase (GGT), α- and π-Glutathione S-Transferase (GST), and albumin were measured on ED presentation, and at 0, 4, 8, and 16 hours, and days 2, 4 and 7 in the ICU in patients after cardiac arrest, sustained or profound hypotension or ruptured abdominal aortic aneurysm. AKI was defined as plasma creatinine increase ≥26.5 μmol/l within 48 hours or ≥50% within 7 days.

**Results:**

In total, 45 of 77 patients developed AKI. Most AKI patients had elevated urinary NGAL, and plasma NGAL and CysC in the period 6 to 24 hours post presentation. Biomarker performance in the ICU was similar or better than when measured earlier in the ED. Plasma NGAL diagnosed AKI at all sampling times, urinary NGAL, plasma and urinary CysC up to 48 hours, GGT 4 to 12 hours, and π-GST 8 to 12 hours post insult. Thirty-one patients died or required dialysis. Peak 24-hour urinary NGAL and albumin independently predicted 30-day mortality and dialysis; odds ratios 2.87 (1.32 to 6.26), and 2.72 (1.14 to 6.48), respectively. Urinary NGAL improved risk prediction by 11% (IDI_event_ of 0.06 (0.002 to 0.19) and IDI_non-event_ of 0.04 (0.002 to 0.12)).

**Conclusion:**

Early measurement in the ED has utility, but not better AKI diagnostic performance than later ICU measurement. Plasma NGAL diagnosed AKI at all time points. Urinary NGAL best predicted mortality or dialysis compared to other biomarkers.

**Trial registration:**

Australian and New Zealand Clinical Trials Registry ACTRN12610001012066. Registered 12 February 2010

## Introduction

Reliance on plasma creatinine delays acute kidney injury (AKI) diagnosis [1,2]. Novel injury biomarkers may enable earlier diagnosis. However, biomarker performance also depends on the interval between insult and time of measurement [3,4]. In the trial, early intervention in acute renal failure (EARLYARF) [5], triaging to intervention within 6 h of admission to the ICU by the urinary tubular enzymes, alkaline phosphatase (AP) and γ-Glutamyl Transpeptidase (GGT) resulted in many false negatives. This occurred because this combination of biomarkers had a short temporal profile with peak urinary concentrations before 12 h after injury and the window of opportunity for diagnosis had been exceeded in many subjects, despite rapid processing in the ICU. Apart from one study of plasma and urinary cystatin C [6] little is known about the temporal profiles of candidate AKI biomarkers of patients presenting to the emergency department (ED). While studies have examined biomarker diagnostic performance in the ED [7,8], subsequent performance at later time points has not been assessed.

We evaluated the temporal profile of plasma creatinine and AKI biomarkers in high-risk critically ill patients, who presented soon after a probable hypoperfusion insult to the kidney. The temporal profiles of biomarker performance in AKI diagnosis and mortality prediction were investigated. We hypothesised that earlier measurement (in the ED) would enhance clinical utility compared with later measurement in the ICU.

## Methods

The EDAKI study was a prospective observational study investigating the utility of plasma and urinary biomarkers from admission to the ED throughout the ICU stay, in patients at high risk of developing AKI. The study was approved by The Upper South A Regional Ethics Committee (URA/09/09/062) and registered under the Australian and New Zealand Clinical Trials Registry (ACTRN12610001012066) and adhered to the Declaration of Helsinki. Patients included were admitted to the ICU at Christchurch Hospital following cardiac arrest, sustained or prolonged hypotension or probable ruptured abdominal aortic aneurysm (AAA). Sustained hypotension was defined as systolic blood pressure <90 mmHg or mean arterial pressure <65 mmHg for 60 minutes after 1 L of intravenous fluid. Profound hypotension was defined as systolic blood pressure <60 mmHg of any duration. Exclusion criteria were: under 16 years old, moribund (not expected to survive 24 h), likely to be discharged within 24 h, drug overdose, treatment limitation order, absent urinary catheter within 4 h of ED admission, already on dialysis, longer than 4 h since admission to the ED, or patients having had inter-hospital transfer unless arrival in the ED occurred less than 3 h after the initiating event, and the patients had not already been admitted to the ICU. Informed consent was obtained from patients, or a family member. Here we present the primary analysis of this study. Partial results for some patients (after cardiac arrest) have been published previously to illustrate an analysis of combining volume and creatinine kinetics [9].

First urine samples were collected during urinary catheterisation in the ED. Routine plasma samples taken on presentation to the ED were retrieved for additional assay. Both plasma and urine samples were collected upon admission to the ICU, and at 4, 8, and 16 h post ICU admission, and at 2, 4, and 7 days. Plasma samples were assayed for creatinine, neutrophil gelatinase-associated lipocalin (NGAL) and cystatin C (CysC), and urine samples were assayed for alkaline phosphatase (AP), γ-glutamyl transpeptidase (GGT), NGAL, α- and π-glutathione S-transferase (GST), CysC, and albumin. Plasma and urinary creatinine were measured by the Jaffe reaction on an Architect c8000 analyzer using Abbott reagents (Abbott Laboratories, Abbott Park, IL, USA). Urinary AP was measured by p-nitrophenol rate reactions and GGT by γ-glutamyl-p-nitroanilide rate on the Architect c8000 analyzer using Abbott reagents (Abbott Laboratories, Abbott Park, IL, USA). Urinary α-GST and π-GST assays were performed by Argutus Medical using enzyme immunoassays (Argutus Medical Alpha GST EIA-BIO-91 and Pi GST EIA-BIO85, Argutus Medical, Dublin, Ireland). Plasma NGAL was analysed using the Triage® NGAL test (Biosite, San Diego, CA, USA). Urinary NGAL was analysed using The Architect Urine NGAL assay (Abbott Diagnostic, Wiesbaden, Germany). Plasma and urinary CysC were measured on a BNII nephelometer (Dade Behring, GmbH, Marburg, Germany) by particle-enhanced nephelometric immunoassay. For AKI outcomes biomarkers were not normalised to urinary creatinine as we have previously shown that for AKI outcomes normalisation reduces the area under the receiver operator characteristic curve (AUC) [10]. Conversely, we showed that for mortality and need for dialysis outcomes normalisation to urinary creatinine improves the AUC, therefore, for the prediction of mortality and dialysis outcomes, we normalised the urinary biomarkers to urinary creatinine.

For each patient, the time of commencing renal hypoperfusion was adjudicated from the time of the ambulance call (for cardiac arrest), or when symptoms of severe shock, such as mental obtundation or hypotension, were documented (for hypotension), or the time of onset of abdominal pain (for suspected ruptured AAA). Baseline plasma creatinine was determined from a chart review, according to the hierarchy: (1) pre-admission creatinine within 7 days to 1 year prior to hospital admission; (2) creatinine at 30-day follow up; (3) hospital discharge creatinine; and (4) creatinine value on admission. AKI was diagnosed using the plasma creatinine criteria, kidney disease improving global outcomes (KDIGO): an increase of more than 26.5 μmol/l (0.3 mg/dl) within 48 h, or a relative increase of more than 50% above baseline within 7 days of admission [11]. Urine output was not used to classify AKI because it was not measured during the early course of the disease in the ED and because of its poor prognostic ability for meaningful clinical outcomes [12,13]. Severity of illness was assessed in each patient during the first 24 h of admission using the acute physiological and chronic health evaluation II (APACHE II) [14]. For the prediction of mortality and dialysis we used the maximum normalised urinary biomarker concentration within 24 h and we evaluated their AKI status during this time (AKI_24_), using as baseline creatinine the pre-admission baseline creatinine where it was known, or the first creatinine measurement in the ED. In this way the prediction is based on clinically relevant variables.

### Statistical analysis

Results were presented as mean ± SD for parametric or median (interquartile range) for non-parametric variables. For continuous variables, differences in two variables were analysed using the independent *t*-test for parametric, or Mann-Whitney *U*-test for non-parametric variables. Differences between three or more variables were determined using one-way analysis of variance (ANOVA) after log transformation where appropriate. Repeated-measures ANOVA was used to compare repeated series of measurements between groups. The multiple imputation technique was used to generate possible values for missing data prior to analysis by repeated-measures ANOVA. Post hoc Fisher’s least significant difference (LSD) analysis was performed for all associations with a *P*-value <0.05 on ANOVA. For categorical variables, differences in proportions were analysed using the Chi-square test or Fisher’s exact test when the sample size was small.

Although an exploratory study, we based the sample size on a power calculation using data from the Early intervention in Acute Renal Failure (EARLYARF) study, in which the false negative rate of the biomarker (GGT × AP >46.3) for detecting AKI was 61%. We determined that, assuming the same AKI incidence rate, 100 patients would provide 85% power at an alpha of 0.05 to detect a false negative rate reduction of 60%. Because of how we selected the cohort we expected a higher AKI incidence rate.

The diagnostic, predictive or prognostic performance of biomarkers was assessed by the AUC. The AUCs were compared by the DeLong method [15]. Multivariable logistic regression was used to calculate odds ratios (ORs) after adjusting for covariates. Variables were included in the multivariable model if the *P*-value was <0.1 on univariate analysis. The additional value of the biomarker to a reference model (comprising variables other than the biomarker identified with *P* <0.1 on univariate analysis) to predict mortality or dialysis was further assessed by the integrated discrimination improvement (IDI) and risk assessment plots [16-18]. The AUC, OR, hazard ratio (HR), and IDI are presented with 95% confidence intervals. Statistical analysis was performed using PASW® version 18.0 (IBM, Somers, NY, USA), PRISM 5.0® (Graph Pad, La Jolla, CA, USA), and MatLab 2011b (MathWorks, Natick, MA, USA).

## Results

**Figure 1:**
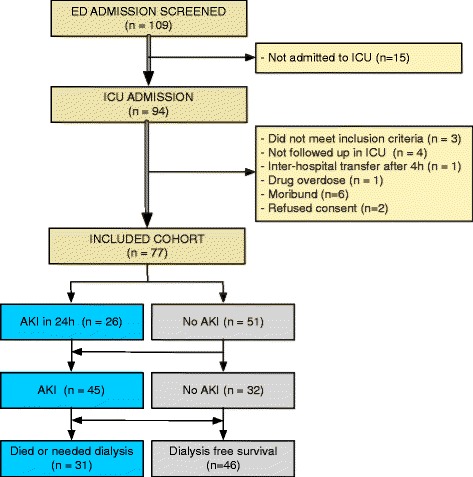
**Patient flow and numbers.**

Between 24 March 2010 and 29 February 2012, 109 patients were recruited. Fifteen patients were excluded because they were not admitted to the ICU. In the ICU, another 17 patients were excluded: 3 did not fit the inclusion criteria, 6 were not expected to survive 24 h, 1 underwent an inter-hospital transfer, 1 a drug overdose, 2 refused to give consent and 4 had no samples taken in the ICU because of clinical priorities (Figure 1). Thus, 77 patients were included in the analysis: 49 (64%) after cardiac arrest, 22 (29%) after sustained hypotension (14 secondary to septic shock), 5 (7%) with ruptured AAA, and one with profound hypotension. Baseline characteristics are presented in Table 1. A total of 45 patients (58%) developed AKI. There were no differences in the demographic profile or baseline co-morbidity between patients with and without AKI. The baseline creatinine used was the pre-admission creatinine in 43 patients, 30-day follow-up creatinine in 18, final creatinine on hospital discharge in 13, and admission creatinine in 3 patients. There was no difference in source of baseline creatinine between those with and without AKI. Amongst survivors, there was no significant difference in the duration of the ICU stay, hospital stay, or mechanical ventilation between those with and without AKI. Of 45 AKI patients, 28 were at severity-stage 1, 8 were stage 2 and 9 were stage 3. A total of 27 patients (35%) died in hospital and 9 (12%) needed dialysis: 31 patients (40%) developed the composite outcome of death or dialysis. The median (interquartile range) between onset of injury and the first ED sample was 0.9 h (0.6 h to 1.1 h) and between the first ED sample and first ICU sample was 1.0 h (0.6 h to 3.2 h).

**Table 1:** **Demographic profiles and clinical characteristics**

**Variables**	**All patients**	**No acute kidney injury**	**Acute kidney injury**	***P*** **-value**
	**(n =77)**	**(n =32)**	**(n =45)**	
Age, years	62 ± 16	62 ± 17	62 ± 15	0.90
Gender, male	57 (74.0)	25 (78.1)	32 (71.1)	0.49
Ethnicity				0.23
- New Zealand European	61 (81.8)	29 (90.6)	34 (75.6)	
- Maori	9 (11.7)	3 (9.4)	6 (13.3)	
- Asian	1 (1.3)	0 (0)	4 (8.9)	
- Others	4 (5.2)	0 (0)	1 (2.2)	
Weight, kg	80 ± 17	80 ± 13	80 ± 20	0.97
Height, cm	171 ± 10	170 ± 8	171 ± 11	0.99
APACHE II score	19 ± 5	19 ± 5	20 ± 5	0.32
Baseline plasma creatinine, μmol/L	92 (74 to 107)	93 (83 to 104)	88 (70 to 109)	0.21
Baseline creatinine selection				0.28
- Pre-hospital (7 to 365 days)	43 (55.8)	17 (53.1)	26 (57.8)	
- 30-day follow up	18 (23.4)	8 (25.0)	10 (22.2)	
- Hospital final	13 (16.9)	7 (21.8)	6 (13.4)	
- First Emergency Department	3 (3.9)	0 (0)	3 (6.7)	
Inclusion criteria				0.01
- Cardiac arrest	49 (63.6)	27 (84.4)	22 (48.9)	
- Sustained hypotension	22 (28.6)	5 (15.6)	17 (37.8)	
- Ruptured abdominal aortic aneurysm	5 (6.5)	0 (0)	5 (11.1)	
- Profound hypotension	1 (1.3)	0 (0)	1 (2.2)	
Baseline comorbidities				
- Hypertension	26 (33.8)	13 (40.6)	13 (28.9)	0.28
- Congestive cardiac failure	7 (9.1)	3 (9.4)	4 (8.9)	0.94
- Ischaemic heart disease	21 (27.3)	11 (34.4)	10 (22.2)	0.24
- Other cardiac diseases	18 (23.4)	8 (25.0)	10 (22.2)	0.78
- Chronic obstructive airways disease	5 (6.5)	1 (3.1)	4 (8.9)	0.31
- Asthma	6 (7.8)	2 (6.2)	4 (8.9)	0.67
- Cerebral vascular accident	13 (16.9)	7 (21.9)	6 (13.3)	0.32
- Diabetes mellitus	11 (14.3)	4 (12.5)	7 (15.6)	0.71
- Kidney impairment	6 (7.8)	4 (12.5)	2 (4.4)	0.19
- Thyroid disease	4 (5.3)	3 (9.4)	1 (2.2)	0.16
- Malignancy	6 (7.8)	3 (9.4)	3 (6.7)	0.66
- Inflammatory diseases	17 (22.1)	5 (15.6)	12 (26.7)	0.25
Clinical Outcomes				
Acute kidney injury within 7 days of admission	45 (58.4)			
Acute kidney injury severity				
- Stage 1	28 (36.3)			
- Stage 2	8 (10.3)			
- Stage 3	9 (11.7)			
Dialysis	9 (11.7)	0 (0)	9 (20.0)	0.007
Hospital mortality	27 (35.1)	9 (28.1)	18 (40.1)	0.28
Composite of mortality or dialysis	31 (40.3)	9 (28.1)	22 (48.9)	0.07
Mechanical ventilation	66 (85.7)	27 (84.4)	39 (86.7)	0.11
Length of mechanical ventilation, h (survivors)	n =40	n =19	n =21	
	44 (26 to 115)	46 (37 to 57)	41 (23 to 234)	0.50
Length of ICU stay, h (survivors)	n =50	n =23	n =27	
	48 (32 to 77)	51 (40 to 73)	47 (19 to 163)	0.51
Length of hospital stay, h (survivors)	n =50	n =23	n =27	
	289 (166 to 409)	289 (194 to 369)	304 (160 to 453)	0.82

Data expressed as mean ± SD, n (%), or median (lower quartile to upper quartile). APACHE II, acute physiological and chronic health evaluation II; CKD, chronic kidney disease; Comparisons of continuous variables between groups were performed using the independent *t*-test for parametric data, and the Mann-Whitney test for non-parametric data (baseline plasma creatinine). Comparisons of categorical data were performed using the Chi-square test.

### Biomarker time courses

**Figure 2:**
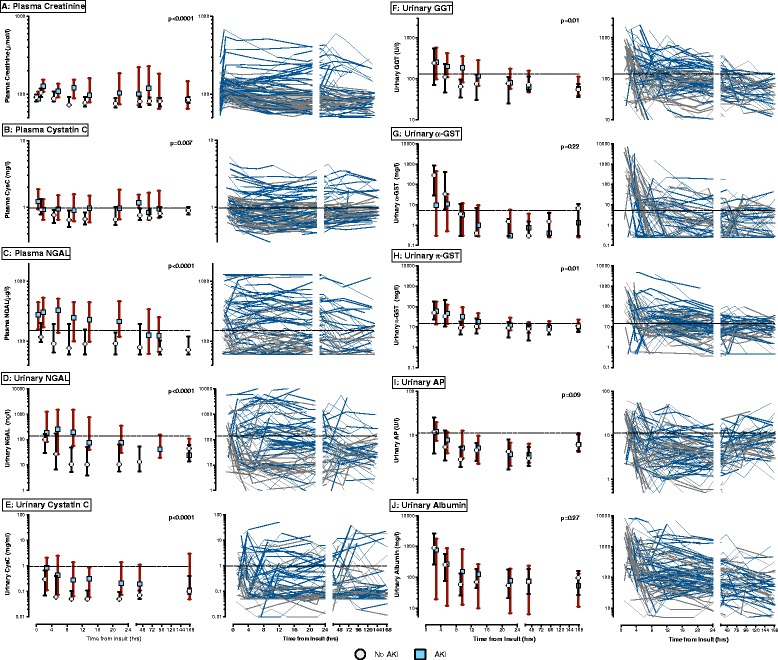
**Temporal profiles of acute kidney injury (AKI) biomarker concentrations in patients with and without AKI.** For each biomarker the graph on the right has individual profiles of AKI patients (blue lines) and non-AKI patients (grey lines). The graph on the left represents median values of AKI patients (blue squares), and non-AKI patients (white circles). Vertical bars cover the interquartile range. The dashed horizontal lines are pre-specified cut points of biomarkers. *P*-values are for comparison of biomarker concentrations between those with and without AKI were performed using repeated-measures analysis of variance after log-transformation. **(A)** Plasma creatinine; **(B)** plasma cystatin C; **(C)** plasma neutrophil gelatinase-associated lipocalin (NGAL); **(D)** urinary NGAL; **(E)** urinary cystatin C; **(F)** urinary gamma-glutamyltranspeptidase (GGT); **(G)** urinary α-glutathione-S-transferase (GST); **(H)** urinary π-GST; **(I)** urinary alkaline phosphatase (AP); **(J)** urinary albumin.

The temporal profiles of the biomarkers between those with and without AKI are shown in Figure 2. Key features are the variation in profiles between patients for all biomarkers, the greater dynamic range (fold-differences) for some biomarkers compared to others, and the greater separation at time points after ED admission (6 to 24 h) between most AKI and non-AKI patients for urinary NGAL, and to a lesser extent plasma NGAL and plasma CysC, compared with other biomarkers. Over the time period, plasma and urinary CysC, and NGAL, and urinary GGT and π-GST concentrations were greater in patients with AKI compared to those without AKI (repeated-measures ANOVA using log values, *P* <0.01). In contrast, there were no differences in urinary α-GST, AP and albumin between those with and without AKI (*P* =0.22, 0.09, and 0.27, respectively).

### Diagnosis of AKI

**Figure 3:**
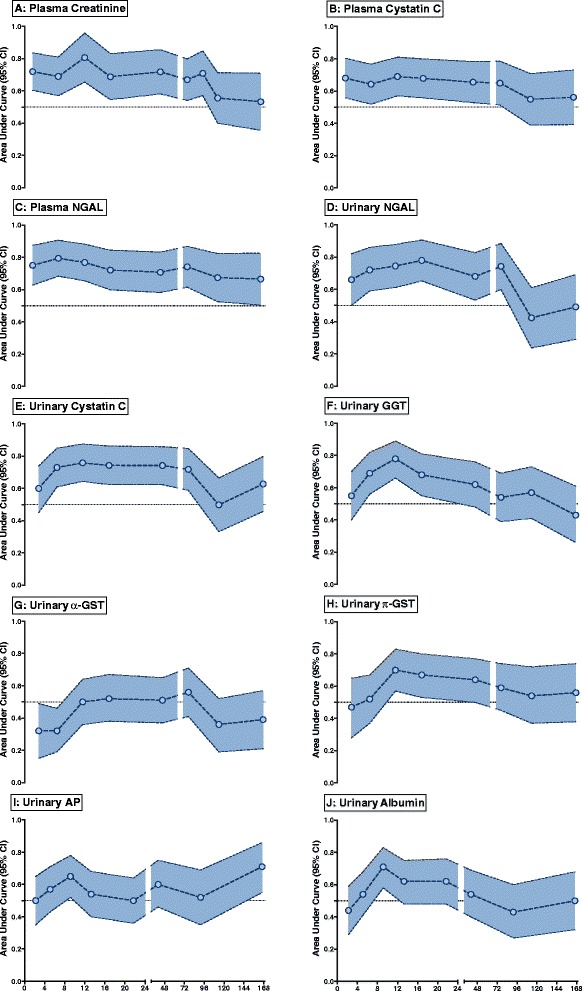
**Time dependence of biomarker performance in diagnosis of acute kidney injury (AKI).** White circles represent the area under the receiver operating characteristic curve (AUC) with 95% confidence interval (shaded areas). An AUC with a confidence interval not overlapping 0.5 was considered diagnostic. **(A)** Plasma creatinine; **(B)** plasma cystatin C; **(C)** plasma neutrophil gelatinase-associated lipocalin (NGAL); **(D)** urinary NGAL; **(E)** urinary cystatin C; (**F**) urinary gamma-glutamyltranspeptidase (GGT); **(G)** urinary α-glutathione-S-transferase (GST); **(H)** urinary π-GST; **(I)** urinary alkaline phosphatase (AP); **(J)** urinary albumin.

The diagnostic performance of the biomarkers as a function of time from renal insult is shown in Figure 3. Plasma NGAL diagnosed AKI at all sampling times. The performance of other biomarkers varied with time from insult; urinary NGAL, plasma and urinary CysC diagnosed AKI within the first 48 h, GGT only between 4 and 12 h, and π-GST from 8 to 12 h.

Biomarker diagnostic performance on presentation to the ED was compared with that on admission to the ICU (Table 2). Plasma CysC and NGAL had similar diagnostic performance at the two sampling times, whereas urinary NGAL, CysC and GGT performed better in the ICU than the ED (*P* <0.05). Diagnostic performance was poor to fair (AUC <0.7) in all cases except plasma NGAL, urinary NGAL and urinary CysC, for which the AUC exceeded 0.7. Urinary π-GST, AP and albumin were not diagnostic of AKI. α-GST was diagnostic of AKI, but at lower concentrations (an AUC of 0.32 means 1/α-GST had an AUC of 0.68).

**Table 2:** **Emergency Department versus initial ICU performance of functional and structural biomarkers in acute kidney injury diagnosis**

**Biomarkers**	**Emergency Department**	**ICU**	***P*** **-value**
Plasma creatinine	0.72 (0.61 to 0.83)	0.69 (0.57 to 0.81)	0.15
Plasma cystatin C	0.68 (0.56 to 0.80)	0.64 (0.52 to 0.77)	0.09
Plasma NGAL	0.75 (0.64 to 0.87)	0.80 (0.68 to 0.90)	0.37
Urinary NGAL	0.68 (0.55 to 0.82)	0.77 (0.66 to 0.88)	0.04
Urinary cystatin C	0.60 (0.45 to 0.74)	0.73 (0.61 to 0.85)	0.004
Urinary GGT	0.55 (0.40 to 0.70)	0.69 (0.56 to 0.82)	0.01
Urinary α-GST	0.32 (0.15 to 0.49)	0.32 (0.18 to 0.46)	0.79
Urinary π-GST	0.46 (0.28 to 0.65)	0.52 (0.37 to 0.67)	0.62
Urinary alkaline phosphatase	0.50 (0.35 to 0.65)	0.57 (0.43 to 0.71)	0.06
Urinary albumin	0.44 (0.29 to 0.59)	0.54 (0.40 to 0.68)	0.08

Data expressed as area under curve with 95% confidence interval. NGAL, neutrophil gelatinase-associated lipocalin; GGT, gamma-glutamyltranspeptidase; GST, glutathione-S-transferase.

Sensitivity analysis was conducted excluding patients who were hypotensive secondary to septic shock, as it was thought possible that the 14 patients who were hypotensive secondary to septic shock could have had an initial insult to the kidney many hours prior to the hypotensive episode. This sensitivity analysis resulted in very similar AUCs to those in Table 2 and similar *P*-values for the difference in AUC between ED and ICU entry (data not shown).

### Prediction of mortality or dialysis

A multivariate logistic regression model based on variables measured within the first 24 h was constructed for prediction of mortality or dialysis. This reference model included variables with *P* <0.1 on univariate analysis, namely the presence of AKI_24_ and the modified APACHE II score (APACHE II score minus creatinine score, because creatinine was part of AKI diagnosis). Twenty-six patients had AKI_24_. New models were constructed comprising the reference model variables plus the addition (separately) of each biomarker. Normalised urinary NGAL and albumin remained independently predictive of mortality or dialysis (OR 2.87 (1.32 to 6.26), and 2.72 (1.14 to 6.48), respectively) (Table 3).

**Table 3:** **Multivariable logistic regression for biomarker prediction of mortality or dialysis after addition to the reference model**

**Peak biomarkers within 24 h (log**_**10**_ )*	**Beta coefficient**	**Odds ratio (95% CI)**	***P*** **-value**
Plasma cystatin C	2.56	12.9 (0.51 to 326)	0.12
Plasma NGAL	0.97	2.62 (0.32 to 21.5)	0.37
Urinary cystatin C/urinary creatinine	0.57	1.77 (0.85 to 3.72)	0.13
Urinary NGAL/urinary creatinine	1.05	2.87 (1.32 to 6.26)	0.008
Urinary alkaline phosphatase/urinary creatinine	0.52	1.68 (0.60 to 4.71)	0.32
Urinary GGT/urinary creatinine	0.46	1.59 (0.52 to 4.81)	0.41
Urinary α-GST/urinary creatinine	0.09	1.10 (0.72 to 1.66)	0.66
Urinary π-GST/urinary creatinine	0.63	1.89 (0.94 to 3.79)	0.07
Urinary albumin/urinary creatinine	1.00	2.72 (1.14 to 6.48)	0.02

*Each biomarker was added separately to the reference model. Urinary biomarkers were normalised to urinary creatinine. The reference model includes variables with *P* <0.1 on univariate analysis, namely the modified acute physiological and chronic health evaluation-II score (without creatinine score) and the presence of AKI_24_. NGAL, neutrophil gelatinase-associated lipocalin; GGT, gamma-glutamyltranspeptidase; GST, glutathione-S-transferase.

**Figure 4:**
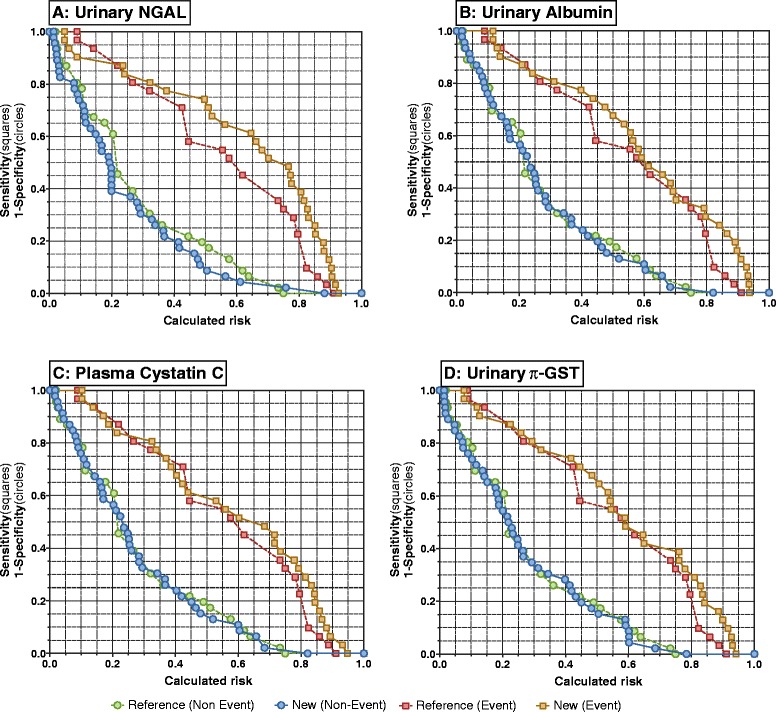
**Risk assessment plots showing the additional value of individual biomarkers compared with the reference model for prediction of the composite of mortality or dialysis.** The reference model included the modified acute physiological and chronic health evaluation (APACHE) II score (without creatinine score), and AKI_24_ (present or absent). The new models comprised the reference model plus peak **(A)** urinary neutrophil gelatinase-associated lipocalin (NGAL), **(B)** urinary albumin **(C)** plasma cystatin C, or **(D)** urinary π-glutathione-S-transferase (GST) within 24 h. Event curves (squares) are sensitivity versus calculated risk. Non-event curves (circles) are 1-specificity versus calculated risk.

The addition of plasma CysC, normalised urinary NGAL, π-GST, and albumin to the reference model improved risk assessment (Table 4 and Figure 4). Urinary NGAL showed the largest improvement (total IDI 0.11): addition of urinary NGAL to the model increased the average calculated risk (IDI_event_) by 0.06 (0.002 to 0.19). The average decrease in calculated risk (that is, for those who did not die or needed dialysis, IDI_non-event_) was 0.04 (0.002 to 0.12). Urinary albumin, π-GST, and plasma CysC showed less improvement.

**Table 4:** **The integrated discrimination improvement index for prediction of mortality or dialysis**

	**IDI event**	**IDI non-event**	**IDI total**	**IS reference model**	**IS new model**	**IP reference model**	**IP new model**
Plasma cystatin C	0.02	0.01	0.03	0.55	0.58	0.27	0.27
	(0.0002 to 0.08)	(0.0001 to 0.047)	(0.0003 to 0.12)	(0.41 to 0.67)	(0.42 to 0.69)	(0.19 to 0.40)	(0.18 to 0.41)
Plasma NGAL	−0.003	0.01	0.01	0.55	0.56	0.27	0.26
	(−0.05 to 0.04)	(−0.02 to 0.06)	(−0.04 to 0.09)	(0.41 to 0.67)	(0.39 to 0.68)	(0.19 to 0.40)	(0.18 to 0.40)
Urinary cystatin C/urinary creatinine	0.02	0.01	0.03	0.55	0.58	0.27	0.27
	(−0.0003 to 0.09)	(−0.0002 to 0.06)	(−0.0005 to 0.15)	(0.42 to 0.68)	(0.42 to 0.69)	(0.19 to 0.40)	(0.19 to 0.42)
Urinary NGAL/urinary creatinine	0.06	0.04	0.11	0.55	0.62	0.27	0.24
	(0.002 to 0.19)	(0.002 to 0.12)	(0.003 to 0.30)	(0.41 to 0.67)	(0.45 to 0.76)	(0.19 to 0.41)	(0.15 to 0.39)
Urinary AP/urinary creatinine	0.0002	0.0002 (−0.001 to	0.0004	0.55	0.56	0.27	0.28
	(−0.002 to 0.005)	0.003)	(−0.003 to 0.008)	(0.40 to 0.67)	(0.39 to 0.67)	(0.19 to 0.39)	(0.20 to 0.42)
Urinary GGT/urinary creatinine	0.003	0.002	0.005	0.55	0.56	0.27	0.28
	(−0.0006 to 0.04)	(−0.0004 to 0.02)	(−0.0009 to 0.06)	(0.41 to 0.68)	(0.42 to 0.69)	(0.19 to 0.41)	(0.21 to 0.42)
Urinary α-GST/urinary creatinine	0.006	0.004	0.009	0.55	0.57	0.27	0.28
	(−0.0005 to 0.05)	(−0.0003 to 0.03)	(−0.0008 to 0.08)	(0.40 to 0.67)	(0.40 to 0.67)	(0.19 to 0.41)	(0.20 to 0.42)
Urinary π-GST/urinary creatinine	0.02	0.02	0.04	0.55	0.58	0.27	0.26
	(0.0001 to 0.11)	(0.0001 to 0.07)	(0.0002 to 0.17)	(0.40 to 0.67)	(0.43 to 0.70)	(0.19 to 0.41)	(0.18 to 0.41)
Urinary albumin/urinary creatinine	0.03	0.02	0.06	0.55	0.60	0.27	0.25
	(0.0006 to 0.12)	(0.0006 to 0.080)	(0.0009 to 0.19)	(0.42 to 0.68)	(0.44 to 0.71)	(0.19 to 0.40)	(0.18 to 0.38)

Data expressed with 95% confidence interval. Reference model: acute physiological and chronic health evaluation-II score (without creatinine), and the presence of AKI_24_ both within 24 h of admission. New model: addition of maximal individual biomarker level within 24 h of admission to the reference model. IDI, integrated discrimination improvement. IDI event, IDI: improvement in risk prediction model for mortality or dialysis; IDI non-event, improvement in risk prediction model for no mortality or dialysis; IP, integrated specificity, that is, differences in average specificity of reference and new model; IS, integrated sensitivity, that is, differences in average sensitivity of reference and new model; NGAL, neutrophil gelatinase-associated lipocalin; AP, alkaline phopshatase; GGT, gamma-glutamyltranspeptidase; GST, glutathione-S-transferase.

## Discussion

Contrary to our hypothesis, earlier biomarker measurement in the ED did not improve prediction of AKI. Plasma cystatin C and NGAL and urinary NGAL all diagnosed AKI in the ED and maintained that diagnostic utility for at least 24 h. Urinary cystatin C and GGT were only diagnostic in the ICU, and in the case of GGT only for a short period of time. The urinary concentrations of GGT, AP, and GST decreased rapidly with little differentiation between those with and without AKI. This highlights that these biomarkers are markers of very early injury, and do not correlate well with change in glomerular filtration rate (GFR). Indeed, the main driving forces of their profiles are the reservoir of available biomarker, which may be released upon injury to the brush border [19]. Albumin decreased similarly with time, although perhaps less rapidly in patients with AKI. Urinary NGAL and to a lesser extent, plasma NGAL and CysC, illustrated distinct elevation in most AKI patients over that of non-AKI patients during the 24 h following ED presentation. For plasma CysC, this reflects the loss of GFR and shorter half-life compared with plasma creatinine. Both plasma and urinary NGAL concentrations are partly affected by loss of GFR as well as tubular injury resulting in upregulation of NGAL and release into both the urine and plasma [19,20]. The differences in temporal profiles highlights the importance of choosing the right temporal window in which to measure these biomarkers. Peak urinary NGAL and albumin within 24 h of presentation were independently predictive of mortality after adjustment for AKI status and illness severity score (APACHE).

A low concentration of urinary α-GST in the ED and ICU was also diagnostic of AKI. This may reflect that α-GST was released even more rapidly than the other biomarkers and was depleted by the time of measurement in the ED. Urinary GGT, α-GST and π-GST were elevated in the ED for nearly all patients. Compared to the cut points for prediction of dialysis need or 30-day mortality determined from the EARLYARF trial [21] GGT was 2 to 4 times greater, α-GST 100 to 1,000 times greater, and π-GST 10 to 100 times greater, regardless of whether subjects had AKI or not, suggesting that these tubular enzymes are very sensitive to renal hypoperfusion, with early release even after mild injury. The significance of an increase in tubular enzymes remains to be tested in a larger patient cohort. As these assays are cheap and rapidly performed, their utility in detecting a milder form of injury remains a potential area for exploration.

Albumin excretion was high in patients without AKI, perhaps because of endothelial dysfunction secondary to inflammation, a known consequence and independent predictor of mortality in critically ill patients [22,23]. Albumin remained increased longer in AKI, possibly due to proximal tubular injury, which reduces albumin reabsorption [24,25].

Induced, biomarkers such as urinary NGAL, and freely filtered biomarkers such as CysC, increase following injury to the proximal and distal tubules. Urinary NGAL is expressed in the distal tubules and collecting duct. NGAL expression in the distal tubules is upregulated in AKI. NGAL is also absorbed into the circulation and filtered later by the glomerulus [26,27]. Reabsorption of filtered NGAL along with other low molecular-weight proteins is impaired in the presence of proximal tubular injury, further increasing urinary excretion [28,29]. Urinary CysC is freely filtered in the glomerulus, and similarly reabsorbed by the proximal tubule. In the presence of proximal tubule injury, reabsorption of CysC is similarly impaired, leading to increased urinary excretion [30]. These biomarkers are released early following insult, and their concentration remains high for a longer duration [4,31]. Their utility in diagnosis of AKI and prediction of mortality has been documented in many studies across different population groups [32-36].

The differences in the response of each biomarker to injury may result from differences in duration of decreased kidney perfusion, or the effect of compensatory mechanisms or intervention. We postulate that a shorter duration of kidney hypoperfusion will result only in tubular enzyme release, whereas a longer duration of hypoperfusion will upregulate the other biomarkers and result in increases of both tubular enzymes and induced or filtered biomarkers. This postulate is supported by the longer duration before return of spontaneous circulation in the sub-cohort of cardiac arrest patients (n =49) who showed increases in both tubular enzymes and induced or filtered biomarkers, compared to those with only increased tubular enzymes (23 ± 13 minutes versus 14 ± 7 minutes, *P* =0.04). Hence, patients with increased tubular enzymes and increased induced or filtered biomarkers may be assumed to have experienced more severe structural injury, compared to those with only tubular enzymuria. Nevertheless, the evaluated biomarkers facilitated in identifying those with tubular cell injury.

We suggest that because of the inherent limitations in using plasma creatinine as a gold standard, the performance of structural biomarkers is best assessed against a meaningful clinical outcome, such as mortality or dialysis. Based on our previous study urinary biomarkers normalised to urinary creatinine are most likely to be of prognostic value [10]. Normalised urinary NGAL and albumin were independently predictive of mortality or dialysis. A 10-fold increase in urinary NGAL or albumin increased the odds of mortality or dialysis by approximately 3-fold. Similarly, Siew *et al.* showed that urinary NGAL was independently predictive of mortality or dialysis in a study of 451 critically ill patients [36]. Albuminuria has also been shown to predict mortality in critically ill patients [22,23].

Both urinary NGAL and albumin improved the clinical model incorporating the APACHE II score and AKI in the prediction of mortality or dialysis. Plasma CysC, and urinary π-GST improved the risk prediction model to a lesser extent than urinary NGAL. Of interest is how much the improvement of risk assessment is clinically important. The question remains as to whether the average increase in risk of 6% identified by addition of urinary NGAL in those who died or were dialysed (IDI_event_), and the average reduction in risk of 4% in those who survived or were dialysis-free (IDI_non-event_), is sufficient to warrant investment in this additional test? Will the indication of structural injury to the kidney change practice? The answer to these questions will only emerge as clinicians engage with the new biomarkers, and learn to use them in high-risk cohorts at the appropriate times.

### Study strengths and limitations

Unique aspects of this study were that it (1) examined critically ill patients at high risk of developing AKI secondary to significant kidney hypoperfusion; (2) captured patients very soon after insult; and (3) measured multiple biomarkers repeatedly from the earliest practicable time point following insult. In the 14 patients with hypotension secondary to septic shock, the onset of sepsis may have decreased filtration prior to the hypotensive event recorded as the time of insult. A sensitivity analysis excluding these patients resulted in only minor differences to the AUC and no differences in the comparison of the AUC for any of the biomarkers for the ED versus the ICU. A lower-than-expected recruitment rate plus interruption to recruitment due to a major earthquake in Christchurch meant the final study numbers after 2-years of recruitment were lower than expected (100). This slightly reduced the power of the study to detect differences in biomarker performance between the ED and ICU. The dilution of creatinine is known to change the diagnosis of AKI in some patients [9,37]. In this analysis we did not adjust the creatinine for fluid balance. Therefore, as fluid balance in the ICU is usually positive, the prevalence of AKI may be overestimated.

## Conclusion

Overall in this cohort of patients at high risk of AKI and with well-documented timing of renal hypoperfusion, plasma NGAL, CysC and urinary NGAL best diagnosed AKI in the ED and continued to perform best over 24 h. The diagnostic performance of six of nine candidate AKI biomarkers (plasma CysC and NGAL, urinary α-GST, π-GST, AP and albumin) were equivalent when measured in the ED or in the ICU. The remaining biomarkers (urinary NGAL, CysC, and GGT) better diagnosed AKI at the later time point on entry to the ICU. The evaluated biomarkers, GGT and AP, facilitated detection of tubular cell injury in the ED, but without apparent diagnostic utility. Peak urinary NGAL within 24 h of admission best predicted the composite outcome of mortality or dialysis requirement.

## Key messages

- AKI injury biomarkers have utility in the ED, but are optimal a few hours later in the ICU
- Urinary NGAL, plasma NGAL and CysC can help diagnose AKI in the ED
- The optimum temporal window for urinary NGAL-, plasma NGAL- and CysC-guided diagnosis is approximately 6 to 24 h following ED presentation
- Tubular cell injury is evident in the ED in most patients shortly after renal insult, but poorly predicts subsequent changes in creatinine

## Abbreviations

AAA: abdominal aortic aneurysm
AKI: acute kidney injury
ANOVA: one-way analysis of variance
AP: alkaline phosphatase
APACHE II: acute physiological and chronic health evaluation II
AUC: area under the receiver operator characteristic curve
CysC: Cystatin C
ED: Emergency Department
GFR: glomerular filtration rate
GGT: gamma-glutamyltranspeptidase
GST: glutahione-S-transferase
IDI: integrated discrimination improvement
NGAL: neutrophil gelatinase-associated lipocalin
OR: odds ratio
